# Influence of Porous Spherical-Shaped Hydroxyapatite on Mechanical Strength and Bioactive Function of Conventional Glass Ionomer Cement

**DOI:** 10.3390/ma10010027

**Published:** 2017-01-03

**Authors:** Szu-Yu Chiu, Yukari Shinonaga, Yoko Abe, Kyoko Harada, Kenji Arita

**Affiliations:** 1 Department of Pediatric Dentistry, Graduate School of Dentistry, Osaka Dental University, 8-1, Kuzuhahanazono-cho, Hirakata, 573-1121 Osaka, Japan; 2 Department of Pediatric Dentistry, Osaka Dental University, 1-5-17, Otemae, Chuo-ku, 540-0008 Osaka, Japan; sinonaga@cc.osaka-dent.ac.jp (Y.S.); abe-y@cc.osaka-dent.ac.jp (Y.A.); kyoko-w@cc.osaka-dent.ac.jp (K.H.); arita-k@cc.osaka-dent.ac.jp (K.A.)

**Keywords:** glass ionomer cement, improvement, hydroxyapatite, cellulose, compressive strength, fluoride release, mineral release

## Abstract

Glass-ionomer-cement (GIC) is helpful in Minimal Intervention Dentistry because it releases fluoride ions and is highly biocompatible. The aim of this study is to investigate the mechanisms by which hydroxyapatite (HAp) improves the mechanical strength and bioactive functioning of GIC when these materials are combined to make apatite ionomer cement (AIC). A conventional GIC powder was mixed with porous, spherical-HAp particles (HApS), crystalline HAp (HAp200) or one of two types of cellulose. The micro-compressive strengths of the additive particles were measured, and various specimens were evaluated with regard to their compressive strengths (CS), fluoride release concentrations (fluoride electrode) and multi-element release concentrations. The AIC was found to release higher concentrations of fluoride (1.2 times) and strontium ions (1.5 times) compared to the control GIC. It was detected the more release of calcium originated from HApS than HAp200 in AIC. The CS of the AIC incorporating an optimum level of HAp was also significantly higher than that of the GIC. These results suggest that adding HAp can increase the release concentration of ions required for remineralization while maintaining the CS of the GIC. This effect does not result from a physical phenomenon, but rather from chemical reactions between the HAp and polyacrylic acid of GIC.

## 1. Introduction

Based on the International Caries Detection and Assessment System (ICDAS), a new caries management process has been established, known as the International Caries Classification and Management System (ICCMS™) [1]. This is a detection and assessment approach that classifies the stages of caries progression, and suggests that only the soft dentin should be removed in the case of deep cavities (ICDAS Code 5–6), and that the tooth structure should not be removed in the case of enamel decalcification (Code 0–2) [2]. One material that is recommended in both scenarios is glass polyalkenoate cement [2,3], as named by the International Organization for Standardization (ISO), and is also well known as glass ionomer cement (GIC).

Because GIC releases fluoride ions [4] and exhibits good biocompatibility with pulp tissue [5,6,7], strong chemical bonding with tooth structures and a low thermal expansion coefficient similar to that of human teeth [8,9,10], it has been widely used as a dental material for some time now. Due to its antimicrobial [11,12] and remineralization [4] effects, GIC has been recommended for use in the Atraumatic Restoration Technique (ART) [13,14]. However, there have been some concerns regarding the inferior physical strength of GIC [15]. To overcome this disadvantage, many researchers have explored the addition of various reinforcing materials to GIC, such as Ag alloy powder, Ag-sintered glass and bioactive glass [16,17,18]. Although these approaches have increased the strength of GIC, they also tend to reduce its ability to release fluoride ions [17,18,19]. In our previous study, we found that the addition of hydroxyapatite (HAp) to GIC can increase its flexural strength while maintaining its compressive strength and simultaneously enhancing fluoride ion release [20,21,22]. Porous, spherical HAp particles were found to be the most effective in this regard [23]. This novel material, which we term apatite ionomer cement (AIC), has also exhibited high antibacterial activity [24].

The mechanisms by which AIC releases more fluoride and is strengthened remain unclear. In the present work, in order to assess these theories, we chose two different types of microcrystalline cellulose that are widely used as pharmaceutical additives, are typically unreactive with other materials, and have similar characteristics and particle morphologies to porous, spherical HAp. Using different ratios of these materials, we compared the fluoride ion release concentration and compressive strengths of the resulting formulations to those of conventional GIC and to AIC.

The aim of this study was to elucidate the mechanisms by which porous, spherical HAp improves the mechanical strength and bioactive functioning of GIC. Our null hypotheses were that the addition of porous, spherical HAp has no effect on the mechanical strength and multi-element release ability of GIC, and that the mechanical strength and multi-element release ability of GIC is unaffected by the addition of cellulose.

## 2. Materials and Methods

### 2.1. Experiment I: The Effects of Varying the Conditions Used to Combine the Additives with GIC

#### 2.1.1. Preparation of Specimens

A conventional, chemically-cured GIC intended for pit and fissure sealing (Fuji III^®^, GC Corp., Tokyo, Japan) was used in this study. Both Fuji III powder, composed of fluoro-aluminosilicate glass, containing aluminum (Al), silicon (Si), and phosphorus (P), as well as strontium (Sr) substituted for calcium (Ca), and Fuji III liquid, containing polyacrylic acid, polybasic carboxylic acid and water, were used in all control and experimental groups. The compositions of the control and experimental groups are summarized in Table 1. AIC samples (AICS group) were made by adding porous, spherical shaped HAp particles (HApS; Taihei Chemical Industrial Co., Ltd., Osaka, Japan) to the Fuji III. Additional comparison specimens were made with two types of microcrystalline cellulose that have similar morphological characteristics to those of the HApS particles. These were a porous material with rod-shaped particles (CEOLUS UF-711, Asahi Kasei Chemicals Co., Tokyo, Japan) and a material with spherical particles (CELPHERE CP-203, Asahi Kasei Chemicals Co., Tokyo, Japan). The groups added the UF-711 and CP-203 particles to GIC were called UFC and CPC, respectively.

In Experiment I, two sets of conditions (A and B) were applied to evaluate the effects of varying these parameters while combining the additives. In both conditions, 1.0 g of the Fuji III powder was used as the control, termed GIC I, and was mixed with 0.83 g of the Fuji III liquid, for a P/L value of 1.2, according to the manufacturer’s recommendations.

Condition set A was employed to generate a group of samples termed AICS-A, and used a quantity of HApS determined based on preliminary studies. In this group, 0.24 g of HApS was combined with 1.0 g of the Fuji III powder. In the case of groups UFC-A and CPC-A, 1.0 g of the Fuji III powder was combined with 0.24 g of the UF-711 or CP-203 powder, respectively.

Condition set B produced the AICS-B group, in which 0.76 g of the Fuji III powder was combined with 0.24 g of the HApS. To maintain a constant additive volume, the UFC-B and CPC-B specimens were made with quantities of UF-711 and CP-203 that had the same volume (0.72 cm^3^) as 0.24 g of the HApS.

#### 2.1.2. Morphological and Strength Analyses of Additives

The powder additives were sputter-coated with osmium using a plasma multi-coater (PMC-5000, MEIWAFOSIS Co., Ltd., Tokyo, Japan) and then observed via scanning electron microscopy (SEM, S-4800, Hitachi High-Technologies Co., Tokyo, Japan). The specific surface areas of the additives were determined by the nitrogen gas adsorption method and the multi-point Brunauer-Emmett-Teller (BET) technique (TriStar-II 3020, Micromeritics Co., Norcross, GA, USA). Micro-compressive strength analysis of the additive particles was performed using a micro-compression instrument (MCT-510, Shimadzu, Kyoto, Japan), applying the formula C = 2.8P/πd^2^, where C is the strength, P is the maximum load (N) and d is the diameter (mm) of the particles.

#### 2.1.3. Compressive Strength Test

Six specimens in each group were prepared by mixing the powder and liquid portions at room temperature and transferring each mixture into a stainless steel split mold (4 mm in diameter and 6 mm in height) using a syringe. Each mixture was held in the mold at 37 °C and 100% relative humidity for 58 min, after which the specimen was removed and stored in artificial saliva (Saliveht™ Aerosol, Teijin Ltd., Osaka, Japan) at 37 °C for the next 23 h. Compressive strength tests were performed with a universal testing machine (AGS-X, Shimadzu Corp., Kyoto, Japan) at a crosshead speed of 1 mm/min.

#### 2.1.4. Fluoride Ion Release Test

Five specimens in each group were prepared by mixing the powder and liquid portions at room temperature and then transferring each mixture into a polyethylene split mold (10 mm in diameter and 2 mm thick). The mold was covered with celluloid strips and a slide glass and subjected to a load of 500 g for 10 min. The disc-shaped specimen was then gently removed from the mold and heated in an incubator at 37 °C and 100% relative humidity for 50 min. Each specimen was subsequently attached to a cotton thread and immersed in 18 mL of deionized water at 37 °C. Refer to our previous studies [21,23,24], the period of measurement was decided for five days. During these trials, each disc was removed from the water and washed with 2 mL of deionized water over the immersion water every 24 h for five days, such that the 2 mL wash water was combined with the original 18 mL water. A 2 mL quantity of total ionic strength adjustment buffer solution (TISAB III, Thermo Fisher Scientific, Beverly, MA, USA) was then added to the combined 20 mL water sample and the fluoride ion concentration in the sample was determined using a fluoride electrode (6561-10C, HORIBA Ltd., Kyoto, Japan) connected to an ion analyzer (D-53, HORIBA Ltd., Kyoto, Japan). Each fluoride release is reported in the form of fluorine mass released per unit sample surface area (μg/cm^2^).

### 2.2. Experiment II: The Effects of HAp on the Mechanical Strength and Various Ion Release Properties of AIC

#### 2.2.1. Preparation of Specimens

**Figure 1:**
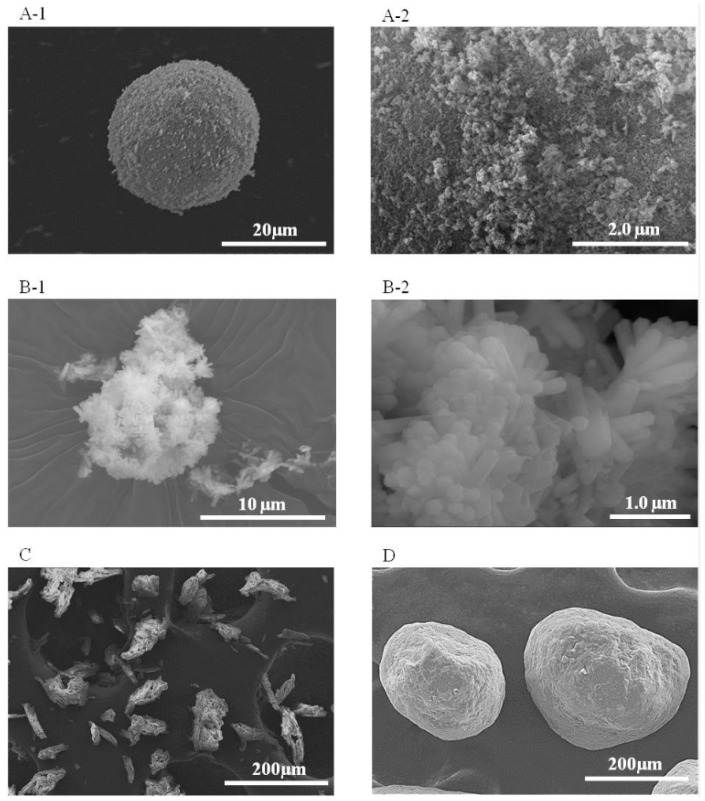
SEM images of the four particulate additives: (**A-1**) a HApS particle; (**A-2**) the surface of a HApS particle; (**B-1**) a HAp200 particle; (**B-2**) the surface of a HAp200 particle; (**C**) a UF-711 particle; and (**D**) a CP-203 particle.

**Figure 2:**
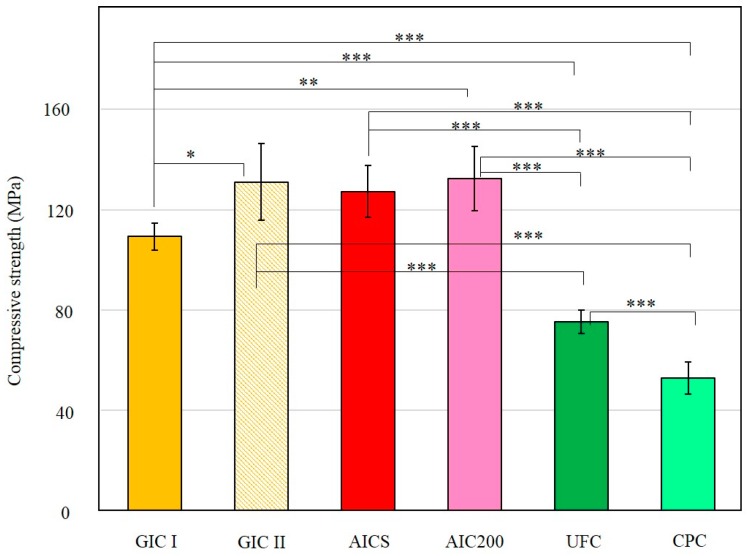
After stored in artificial saliva at 37 °C for 23 h, the compressive strength data obtained from the Experiment II (*n* = 6/group); * *p* < 0.05; ** *p* < 0.01; *** *p* < 0.001.

The formulations of the control and experimental groups in Experiment II are provided in Table 2. Here, the conditions used when preparing the GIC I, AICS, UFC and CPC specimens were the same as those used to make the corresponding materials during Experiment I—Condition A. The GIC II powder was composed of 1.24 g of Fuji III powder mixed with 0.83 g of Fuji III liquid (P/L = 1.49). All other groups were made using this same P/L value. In addition, a HAp with hexagonal crystals (HAp200, Taihei Chemical Industrial Co., Ltd., Osaka, Japan; Figure 1(B-1,B-2)) was also used as an additive to make another AIC, designated AIC200.

#### 2.2.2. Morphological and Strength Analyses of the Additive

Using the same methods described in Section 2.1.2, the microstructures of the HAp200 particles were observed by SEM, while the micro-compressive strength and specific surface area of the HAp200 were obtained from a report by Arita et al. [21].

#### 2.2.3. Compressive Strength Test

The same method described in Section 2.1.3 was used to determine the compressive strengths of the specimens in Experiment II.

#### 2.2.4. Fluoride Ion Release and Multi-Element Release Tests

The same method described in Section 2.1.4 was used to determine the fluoride ion release concentration from the specimens in Experiment II.

In addition, another five specimens in each group were made using the methods described above for the fluoride release test in Section 2.1.4, and immersed in deionized water at 37 °C for five days. The amounts of Al, Si, P, Ca and Sr released from the specimens were subsequently measured by inductively coupled plasma atomic emission spectroscopy (ICP-AES; ICPS-8100, Shimadzu Co., Kyoto, Japan).

### 2.3. Statistical Analysis

Herein, the data are presented in the form of mean ± standard deviation (S.D.). The data were analyzed by one-way ANOVA and Tukey’s test (KaleidaGraph 4.00, SYNERGY SOFTWARE, Reading, PA, USA), with p values less than 0.05 being considered statistically significant. The confidence interval was set at the 95% confidence level.

## 3. Results

### 3.1. Experiment I: The Effects of Varying the Conditions Used to Combine Additives with the GIC

#### 3.1.1. Morphological Characteristics of Additives

SEM images of the additive particles are shown in Figure 1. The HApS particles were spherical and approximately 20 μm in diameter (Figure 1(A-1)), with numerous nanometer-sized particles around the surface of each larger particle (Figure 1(A-2)). The UF-711 particles had irregular shapes and appeared porous (Figure 1C), while the CP-203 particles were spherical and approximately 150 to 300 μm in diameter (Figure 1D).

The micro-compressive strength and specific surface area values of these materials are shown in Table 3. The HApS particles had an extremely low compressive strength of 0.06 ± 0.061 MPa. The strength of the UF-711 particles could not be measured because of their non-spherical and extremely irregular shapes. The CP-203 particles had a very high micro-compressive strength of 23.49 ± 5.664 MPa and were observed to fracture without being completely crushed.

The specific surface area of the HApS particles was 42.14 m^2^/g, while the UF-711 and CP-203 had values of 1.08 and 0.02 m^2^/g, respectively.

#### 3.1.2. Compressive Strengths of Specimens

The compressive strengths obtained when using Conditions A and B are shown in Table 4. The AICS-A sample had almost 1.2 times higher strength than the GIC I specimens. In addition, the compressive strengths of the UFC-A and CPC-A were significantly reduced compared to those of the GIC I and AICS-A (*p* < 0.001). The compressive strength of the AICS-B was significantly higher than that of the GIC I (*p* < 0.05), and the strengths of the UFC-B and CPC-B were below those of the GIC I and AICS-B (*p* < 0.001).

#### 3.1.3. Fluoride Ion Release Properties

The accumulated amounts of fluoride released from specimens prepared using Conditions A and B are summarized in Table 5. The fluoride ion release of the AICS was significantly higher than those of the GIC I and UFC, while there was no significant difference between the AICS and CPC specimens made using Conditions A and B. In addition, all the Condition B groups had higher fluoride release rates than the Condition A samples.

### 3.2. Experiment II

#### 3.2.1. Morphological Characteristics of the Additives

SEM images of HAp200 particles are shown in Figure 1(B-1,B-2). The HAp200 was evidently composed of hexagonal crystals about 0.5 μm in width and 2 to 3 μm in length, often formed into aggregates approximately 13 μm in diameter.

The micro-compressive strength and specific surface area of HAp 200 have been reported to be 1.54 ± 0.225 MPa and 6.52 ± 0.08 m^2^/g [21]. HAp200 is therefore stronger and less porous than HApS.

#### 3.2.2. Compressive Strengths of Specimens

The results of compressive strength data obtained from the Experiment II are summarized in Figure 2. The compressive strengths of the GIC II and AIC200, in which the P/L ratio was 1.49, were higher than that of the GIC I (P/L = 1.2). There were no differences between the AICS, GIC I and GIC II. The compressive strengths of the UFC and CPC were also significantly lower than those of the control and the other experimental groups.

#### 3.2.3. Fluoride Ion and Multi-Mineral Release Properties

**Figure 3:**
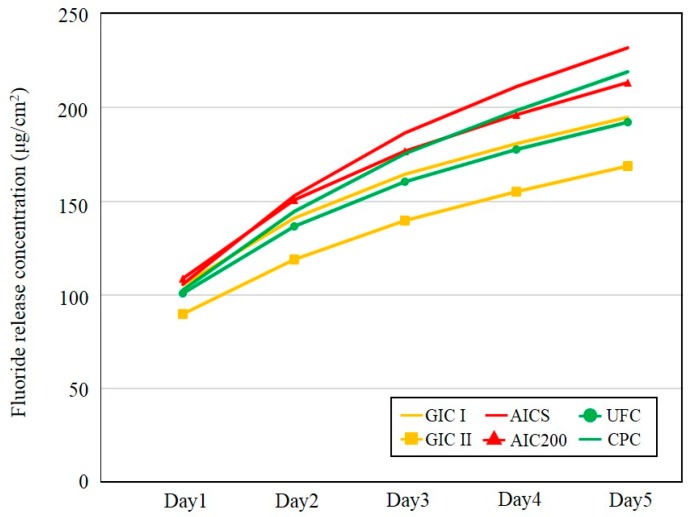
The accumulated amounts of fluoride ions released during Experiment II per unit sample surface area after five days (*n* = 5/group).

The accumulated amounts of the fluoride ion released during Experiment II test are presented in Figure 3 and the results of statistical analyses are shown in Table 6. After five days, the AICS released the highest accumulated amount of fluoride ions, and there was also a statistically significant difference between the AICS and GIC I and GIC II (*p* < 0.05; *p* < 0.001). There was no difference between the AIC200 and AICS, although the AIC200 released a significantly higher level of fluoride than the GIC II (*p* < 0.01).

**Figure 4:**
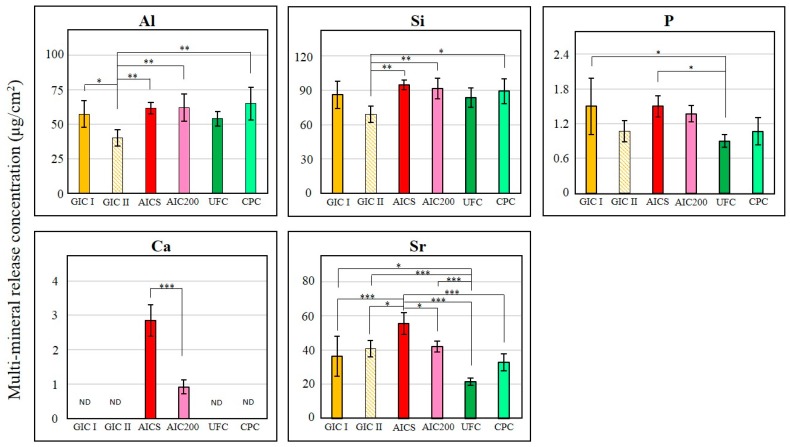
After immersed in deionized water at 37 °C for five days, the quantities of Al, Si, P, Ca and Sr generated during Experiment II per unit sample surface area (*n* = 5/group). ND: not detected. (* *p* < 0.05; ** *p* < 0.01; *** *p* < 0.001).

Figure 4 presents the results of the multi-element release test, showing that the release concentrations of Al and Si were similar in the cases of AICS, AIC200 and CPC, all of which released significantly higher amounts than GIC II (*p* < 0.05 between the concentration of Si released by CPC and GIC II; *p* < 0.01 between others). There were no statistical differences between the GIC I and the experimental groups. The concentration of P released from the UFC was significantly lower (1.7 times lower) than those obtained from the AICS and GIC I. However, the concentrations of Ca ion produced by the AICS were three times higher than AIC200, and the concentrations of Sr ion which also released by the AICS were significantly higher than those from the other groups (approximately 1.3 times higher than GIC II and AIC200; 1.5 times higher than GIC I; 1.7 times higher than CPC; and 2.6 times higher than UFC).

## 4. Discussion

The AIC specimens in our previous studies [20,21,22,23,24] were made by reducing GIC powder and adding HAp powder instead. Because fluoride is contained in the GIC glass powder, it was not clear why the AIC had superior fluoride release properties in spite of a decrease in the amount of GIC powder. The fluoride release from GIC is normally due to an acid–base reaction, with the amount of fluoride released being proportional to the concentration of fluoride in the material [25]. Therefore, the results obtained from these AICs appear contrary to the expected outcome. In Experiment I, we used constant masses of the Fuji III powder (the fluoro-aluminosilicate glass) and Fuji III liquid (the polyacrylic acid) in both the GIC I and in the experimental groups during the Condition A preparation to address this question. The fluoride release data obtained from the Condition A specimens showed that the amount of fluoride released from the AICS-A was significantly higher than from the GIC I, even though all groups had the same mass of the Fuji III powder, which was the only source of fluoride ions (Table 5). These results suggest that HApS could play an important role in increasing the fluoride release based on a reaction between HApS and the GIC matrix or glass core. Moreover, in medical fields, porous HAp has also been studied as a drug delivery system, and it was reported that the microporosity of HAp allowed the slow release of drug [26]. It was considered that HApS acted as the pathway of fluoride ion release due to its porosity. Namely, it is possible that the matrix included fluoride ion originated from GIC glass core infiltrated the pores of HApS in AICS, and the fluoride ions were released out of AICS.

We also looked at the results obtained when adding cellulose materials that were incapable of reacting with the GIC. The fluoride ion releases from the CPC-A and UFC-A were found to be similar to that obtained from the GIC I (Table 5). However, the fluoride release from the CPC-A was not significantly different from that of the AICS-A. This might be explained by considering that the density of the UF-711 powder (0.22 g/cm^3^) was lower than that of the HApS (0.33 g/cm^3^), while the CP-203 was the densest (0.87 g/cm^3^). For this reason, the volume of the CP-203 powder in the Condition A formulation was low compared to that of the UF-711 powder. The release of fluoride ions from the GIC powder is due to a reaction with the GIC liquid [27], so it appears that a greater quantity of the Fuji III liquid was able to react with the Fuji III powder in the CPC specimens. To further verify the relationship between fluoride release and the P/L ratio, and to demonstrate that the fluoride release properties of AICs are not related to the volumes of additives, Condition B was designed. This fabrication process involved using volumes of the UF-711 and CP-203 powders equal to the volume of a 0.24 g quantity of the HApS powder, thereby removing the variations in the volume of additive powder. The fluoride release from the CPC-B was not significantly different from that of the AICS-B, which exhibited increased fluoride ion release properties. In addition, there was no significant difference between the UFC-B and AICS-B. The variation in fluoride release between the UFC and CPC groups may therefore be due to differences in their water absorption characteristics. It appears that the fluoride release properties of the formulations with UF-711 and CP-203 powder added to the GIC might not be related to chemical reactions between the GIC and the celluloses, but rather to physical factors such as the water absorption properties. Further research is still required to identify the regions within the GIC that deteriorate to release fluoride ions, but we can hypothesize that fluoride ion release is not the result of physical degradation of the material. These data suggest that the proportions of the GIC and HAp powders in AIC formulations should not be determined solely on considerations of volume.

Experiment II was designed to confirm the effects of different HAp materials on the mechanical strength and ion release properties of AICs. Arita et al. demonstrated that porous HAp made by grinding HAp with columnar crystal shapes using an automatic ball mill is suitable for use in dental restorative AIC formulations [21]. In our study, a commercial porous HAp (HApS) was selected because of its low cost, and because it can produce an AIC with applications as a restorative and sealing material [23,24]. However, an AIC including this spherical, porous HApS has not yet been compared with materials made with HAp powders with other morphologies. Accordingly, we selected HAp200, which has a typical hexagonal crystal shape. In addition, some researchers have reported that cellulosic fibers improve the mechanical properties of GIC, including its compressive or diametrical tensile strength [28,29]. In our study, celluloses having either porous or spherical characteristics, UF-711 and CP-203, were also compared with HApS. Another control group, GIC II, was made for comparison purposes at the same P/L ratio as the experimental groups. The compressive strength data obtained in Experiment II showed no significant difference between the AICS and AIC200. The HAp200 particles are highly crystalline and exhibit high micro-compressive strength (1.54 MPa) [21], while the strength of HApS is extremely low (0.06 MPa, Table 3). Interestingly, the compressive strength of CPC was significantly lower than that of the GICs and AICs, even though the CP-203 particles had a higher micro-compressive strength (23.49 MPa). These data suggest that there is no benefit in adding reinforcing materials that do not undergo chemical reactions with the GIC. In addition, the HApS particles are breakable and can therefore disperse into the matrix layer [24]. It has also been shown that HAp can act as a drug delivery carrier due to its superior adsorptive properties [30,31]. Moreover, HApS showed the agglomeration of nano-HAp particles in the SEM image (Figure 1(A-2)). Amorphous, not well-crystallized HAp primary particles typically exhibit higher solubility. It has been demonstrated that Ca enhances the formation of the GIC matrix, increasing the surface hardness [32]. Therefore, the dispersion of HApS particle in the matrix leads to a chemical reinforcement effect and increases the compressive strength.

In terms of fluoride ion release properties, there was no significant difference in the fluoride ion release concentrations between AICS and AIC200, although the AICS tended to exhibit higher fluoride ion release compared to the AIC200. GIC glasses contain other elements, such as Al, Si, P and Sr, and these cations can produce a complex phosphate hydrogel matrix [33]. In our previous study, it was observed that AIC samples had a GIC glass core within a polyacrylic acid matrix-gel layer [23,24]. In the present work, the ICP-AES results demonstrated that HApS and HAp200 do not appear to increase the amounts of Al, Si and P ions released from the GIC, nor do they inhibit the original release concentrations. In Fuji III, Sr is added to the polyalkenoate glass instead of Ca, so the Sr detected by ICP-AES originated from the Fuji III powder. In contrast, the Ca came from the HApS or HAp200, so it was not detected in the control groups (GIC I and GIC II) or the two cellulose-added groups (UFC and CPC). The Sr and Ca release concentrations from the AICS were significantly higher than that from the AIC200. Due to its high specific surface area, HApS might be easier to expose to the Fuji III liquid compared to HAp200 which had well-crystalized primary particles. It was considered that HAp increase dissolution kinetics and might lead to an overall larger Ca ion release by the reason of its solubility. It has been reported that a combined Sr-F treatment for softened enamel promotes remineralization and prevents acid demineralization [34]. GIC has been shown to exhibit remarkably high adhesion to tooth surfaces, and the intermediate layer between GIC and dentin contains Ca and P originating from the dentin material HAp [35]. AICs are therefore expected to have superior tooth adhesion properties. Moreover, it is possible that the superior ion release properties of AICs could promote the remineralization of enamel and dentine and form a secondary dentin to prevent caries or secondary caries.

## 5. Conclusions

A porous, spherical HAp was mixed with a conventional GIC. The addition of this HAp improved the fluoride release properties and the compressive strength of the GIC. The use of the spherical HAp also increased the release concentration of Sr and Ca ions compared with the use of highly crystalline HAp. It is anticipated that GICs incorporating porous, spherical HAp could be helpful in the remineralization of tooth substrates and the prevention of caries and secondary caries.

## Figures and Tables

**Table 1:** The compositions of specimens used in Experiment I.

Group		Powders				Liquid	Powder/Liquid (g/g)
		Fuji III (g)	Additives (g)	Total (g)	Proportion of Additives (wt %)	Fuji III (g)	
		[*Volume*(cm^3^)]	[*Volume*(cm^3^)]	[*Volume*(cm^3^)]	[*Volume* %]		
GIC I		1.0	0	1.0	0	0.83	1.2
		[*0.357*]		[*0.357*]			
A	AICS	1.0	0.24 HApS	1.24	19.4	0.83	1.49
					[*66.9*]		
	UFC	1.0	0.24 UF-711	1.24	19.4	0.83	1.49
					[*75.3*]		
	CPC	1.0	0.24 CP-203	1.24	19.4	0.83	1.49
					[*43.5*]		
B	AICS	0.76	0.24 HApS	1	24	0.83	1.2
			[*0.72*]		[*71.4*]		
	UFC	0.76	0.16 UF-711	0.92	17.4	0.83	1.1
			[*0.72*]		[*71.4*]		
	CPC	0.76	0.63 CP-203	1.39	45.3	0.83	1.67
			[*0.72*]		[*71.4*]		

In the two conditions (A and B), 1.0 g of Fuji III powder was used as a control, termed GIC I. **Condition A. Calculated according to weight as in our previous study.** The same weight of GIC powder as used in the control (GIC I) was employed in each experimental group, with the addition of 0.24 g of HApS, UF-711 or CP-203. **Condition B. UF-711 or CP-203 volumes equal to that of 0.24 g of HApS powder.** Each sample was a mixture of 0.76 g of GIC powder with a mass of additive having a volume equal to the volume of 0.24 g of HApS powder.

**Table 2:** The formulations of specimens used in Experiment II.

Group	Powders				Liquid	Powder/Liquid (g/g)
	Fuji III (g)	Additives (g)	Total (g)	Proportion of Additives (wt %)	Fuji III (g)	
GIC I	1.0	0	1.0	0	0.83	1.2
GIC II	1.24	0	1.24	0	0.83	1.49
AICS	1.0	0.24 HApS	1.24	19.4	0.83	1.49
AIC200	1.0	0.24 HAp200	1.24	19.4	0.83	1.49
UFC	1.0	0.24 UF-711	1.24	19.4	0.83	1.49
CPC	1.0	0.24 CP-203	1.24	19.4	0.83	1.49

Based on Condition A in Experiment I, GIC II and AIC200 were also examined. The mass of Fuji III powder in the GIC II specimen was equal to the sum of the powder masses in the experimental groups. The AIC200 was prepared by adding HAp200 to GIC powder following the same method as applied to the other additives.

**Table 3:** The micro-compressive strengths and specific surface areas of the four particulate additives.

Specimen	Micro-Compressive Strength (MPa)	Specific Surface Area (m^2^/g)
HApS	0.06 ± 0.06	42.14 ± 0.08
HAp200	1.54 ± 0.23 [21]	6.52 ± 0.08 [21]
UF-711	-	1.08 ± 0.02
CP-203	23.49 ± 5.66	0.02 ± 0.00

**Table 4:**
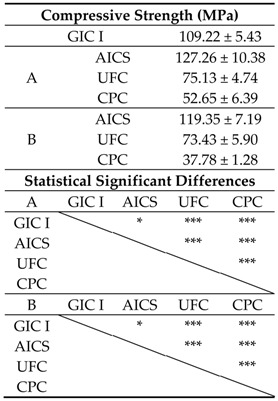
After stored in artificial saliva at 37 °C for 23 h, the compressive strengths of specimens prepared using the two sets of conditions in Experiment I, and the results of statistical analyses. (*n* = 6/group).

* *p* < 0.05; ** *p* < 0.01; *** *p* < 0.001.

**Table 5:**
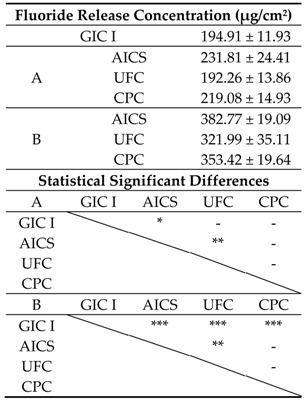
After five days, the accumulated amounts of fluoride ions (per unit sample surface area) released from samples prepared using the two conditions in Experiment I and the results of statistical analyses. (*n* = 5/group).

- *p* > 0.05; * *p* < 0.05; ** *p* < 0.01; *** *p* < 0.001.

**Table 6:**
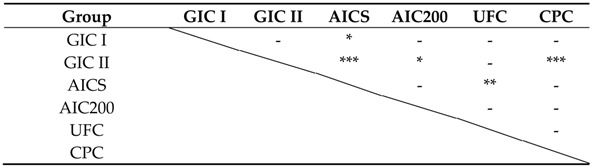
Statistical significance of differences in the accumulated amounts of fluoride released after five days.

- *p* > 0.05; * *p* < 0.05; ** *p* < 0.01; *** *p* < 0.001.
